# Combination of STING Pathway Agonist With Saponin Is an Effective Adjuvant in Immunosenescent Mice

**DOI:** 10.3389/fimmu.2019.03006

**Published:** 2019-12-23

**Authors:** Elena V. Vassilieva, Dahnide W. Taylor, Richard W. Compans

**Affiliations:** Department of Microbiology and Immunology, Emory Vaccine Center, Emory University School of Medicine, Atlanta, GA, United States

**Keywords:** aged mice, influenza vaccine, adjuvant, cGAMP, saponin

## Abstract

There is an urgent need to improve protective responses to influenza vaccination in the elderly population, which is at especially high risk for adverse outcomes from influenza infection. Currently available inactivated vaccines provide limited protection, even when a 4-fold higher dose of the vaccine is administered. Adjuvants are often added to vaccines to boost protective efficacy. Here we describe a novel combination of an activator of the STING pathway, 2′,3′-cyclic guanosine monophosphate–adenosine monophosphate (cGAMP) with a saponin adjuvant, that we found to be highly effective in boosting protective immunity from vaccination in an aged mouse model. Using this combination with a subunit influenza vaccine, we observed that survival of vaccinated 20 month-old mice after lethal challenge increased from 0 to 20% with unadjuvanted vaccine to 80–100%, depending on the vaccination route. Compared to unadjuvanted vaccine, the levels of vaccine-specific IgG and IgG2a increased by almost two orders of magnitude as early as 2 weeks after a single immunization with the adjuvanted formulation. By analyzing phosphorylation of interferon regulatory factor 3 (IRF3) in cell culture, we provide evidence that the saponin component increases access of exogenous cGAMP to the intracellular STING pathway. Our findings suggest that combining a STING activator with a saponin-based adjuvant increases the effectiveness of influenza vaccine in aged hosts, without having to increase dose or perform additional vaccinations. This study reports a novel adjuvant combination that (a) is more effective than current methods of boosting vaccine efficacy, (b) can be used to enhance efficacy of licensed influenza vaccines, and (c) results in effective protection using a single vaccine dose.

## Introduction

The low efficacy of current influenza vaccines is a significant public health problem, which presents an even greater challenge for the aged population because of immunosenescence (1–3). It is estimated that adults 65 and older accounted for 70% of hospitalizations and close to 70,000 deaths, representing 90% of overall influenza-related mortality in the vaccine-eligible population of United States during the 2017–2018 influenza season in spite of the highest vaccination rate within ≥65 age group among all adults (4). Current vaccination strategies for individuals over 65 years of age in the U.S. include administering a 4-fold higher dose of vaccine antigens per strain than is recommended for healthy adults, either alone or in the presence of an adjuvant (5–11). These approaches have been only moderately successful, and there is an unmet need to improve vaccination to protect the aged population which fuels the search for a better adjuvant that is effective in this age group, an objective of the present work. We employed an aged mouse model that has been shown to recapitulate age-related changes in humans (12, 13) including responses to influenza infection and vaccination (14, 15). Several adjuvant formulations have been previously shown to increase protective efficacy of hemagglutinin (HA)-based influenza vaccines in aged mice, but these formulations required use of complex preparation procedures and some included more than one dose of vaccine (16–19). However, influenza vaccine is administered only once during the annual vaccination campaigns. Thus, the objective of the present study was to identify an improved adjuvant formulation potentially compatible with different immunization routes and effective with a single dose of a licensed HA-based influenza vaccine.

An agonist of the intracellular stimulator of interferon genes (STING) pathway, cyclic di-nucleotide a 2′3′-cGAMP, is an experimental vaccine adjuvant (20–23). The signaling cascade triggered by activation of STING (24, 25) leads to production of IFN-β and other cytokines important for innate immunity (26–28) without causing adverse reactions attributed to Toll-like receptor agonists or Alum (22). However, rather high amounts of cGAMP or other cyclic di-nucleotides have been required for adjuvant activity (22, 29, 30). We hypothesized that improving the availability of the cGAMP ligand to the STING receptor in the interior of the immunocompetent cells of aged subjects will increase its adjuvant efficiency. To test this hypothesis, we designed a new approach for delivery of cGAMP in the presence of a membrane-active saponin-based adjuvant, thus combining adjuvantic potential of both compounds. We evaluated the immunogenicity of an influenza subunit vaccine adjuvanted with each component separately or in combination, and assessed the protective efficacy of the adjuvanted vaccine in live virus challenge experiments. To address the effect of advanced age, we also compared the same vaccine formulations in mature adult mice.

## Materials and Methods

### Ethics Statement

All institutional and national guidelines for the care and use of laboratory animals were followed in accordance with and approved by the Institutional Animal Care and Use Committee (IACUC) at Emory University.

### Animals

Female BALB/cAnNCrl mice from Charles River Labs (Wilmington, MA) were used in all experiments. Mice were housed in microisolators with filter tops in a biocontainment level BSL-1 animal facility and subjected to a 12/12-h light/dark cycle and temperature between 20 and 22°C until they reached 4 (adults) or 19 (aged) months of age. For challenge experiments, animals were moved to a BSL-2 facility operating under the same light and temperature conditions.

### Viruses

H1N1 Influenza A/California07/09 virus was obtained from the Centers for Disease Control and Prevention (CDC, Atlanta, GA), grown in MDCK cells and used for hemagglutination inhibition (HAI) titration of sera. The virus was mouse-adapted by serial passage in the lungs of adult BALB/c mice (31) and was used in challenge experiments. The LD_50_ was determined in adult female BALB/c mice using the Reed-Muench calculation method (32).

### Vaccine and Adjuvants

Influenza A (H1N1) 2009 A/California/07/09 H1N1 vaccine was obtained from BEI resources (NR-20347). It was concentrated by ultrafiltration and HA content was determined by SRID assay as previously described (33), using strain specific reagents from the Center for Biologics Evaluation and Research (Kensington, MD). AddaVax, Quil-A, and 2′,3′-cGAMP (cyclic [G(2′,5′)pA(3′,5′)p] were purchased from Invivogen (San Diego, CA). The stock solutions of Quil-A and cGAMP were prepared in 50 mM potassium phosphate buffer, pH 7.4. AddaVax (nanoemulsion produced from 0.5% sorbitan trioleate in 5% squalene oil and 0.5% Tween-80 in 10 mM sodium citrate buffer pH 6.5), 25 μl per dose, was mixed with the same volume of vaccine prior to immunization. Except for the high dose vaccine formulation, the amount of vaccine antigen was 1 μg in all animal experiments. The immunogen was mixed with Quil-A in a vaccine/adjuvant ratio between 1:1 and 1:10 and with cGAMP between 1:1 and 1:5 (wt/wt, μg), as specified for each experiment.

### Immunization, Challenge, Sample Collection, and Analysis

We employed BALB/c mice that were 19 months old at the time of vaccination and are classified as aged (34), as well as 4 month old mature adult mice. Mice were immunized once intramuscularly (IM) by injection (0.05 ml volume, 30-gauge needle) either into the upper quadrant of the hind leg, or intradermally (ID) into depilated dorsal skin (bleb was observed) under xylazine/ketamine anesthesia. Blood samples were collected from the fascial vein on days 7, 14, and 28 post vaccination and analyzed for HAI titers and vaccine-specific immunoglobulins as described previously (35). HAI titers were converted into log_2_ values for statistical analysis. For challenge studies, aged mice were infected with ~300 plaque forming units (pfu) of the mouse—adapted virus, and adult mice received a 10-fold higher dose which was equivalent to 70 × LD_50_. Challenge was performed by intranasal installation of 30 μl of diluted virus under brief isofluorane anesthesia 5.5 weeks after single immunization. Mice were monitored for signs of infection for 2 weeks as previously described (33). The humane endpoint used for euthanasia was 25 % loss of the initial body weight.

### Cell Culture Experiments

HeLa cells and murine embryonic fibroblasts isolated from the wild type (STING +/+) or STING knockout (STING^−/−^) mice with C57BL/6J genetic background (MEFs, a generous gift of Dr. G. Barber) (26), were grown in 48-well plates in DMEM media supplemented with 1% FBS and Penn/Strep antibiotics (Corning, NY). Confluent cells were treated with A/California/07/09 H1N1 vaccine and individual adjuvants or their combination for 1 h at 37°C, after which they were immediately collected on ice into reducing Laemmli sample buffer (BioRad, CA) supplemented with protease inhibitors (Sigma, MO), phosphatase inhibitors (Roche, Germany) and DNAse I (Sigma, MO). Cell lysates were analyzed by SDS-PAGE and western blot, and probed for pIRF3 (from Abcam ab76493 for HeLa and from Cell Signaling D601M #29047 for MEFs) and actin (from Biolegend Direct-Blot #643807 for HeLa and Sigma #A2066 for MEFs) using ECL detection (Thermo Scientific, Il) and BioRad imager software for quantification.

## Results

### cGAMP and Quil-A as Individual Adjuvants in Aged Mice

**Figure 1:**
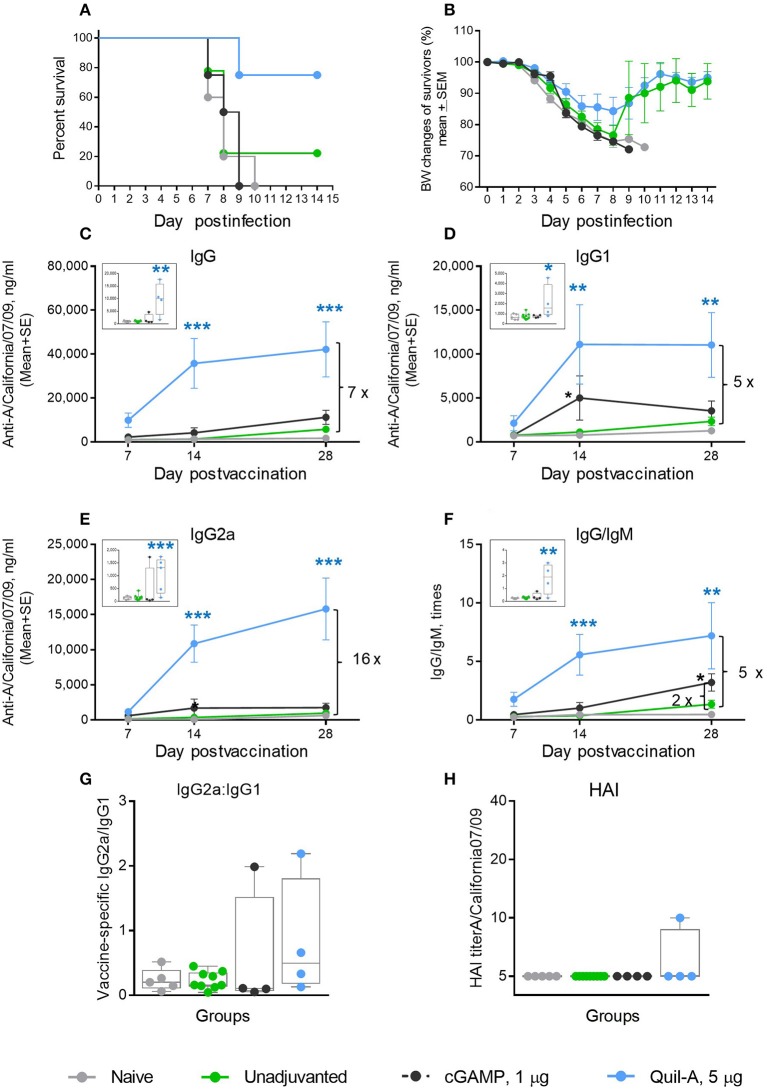
Effect of ID administration of 1 μg of A/California 07/09 (H1N1) vaccine supplemented with either 5 μg cGAMP or 5 μg Quil-A on protective immunity in aged mice. **(A)** Survival and **(B)** weight chart of the surviving mice challenged with mouse-adapted influenza A/California 07/09 H1N1virus; **(C–F)**—time course of vaccine-specific antibody response plotted against day post-vaccination: IgG **(C)**, IgG1 **(D)**, IgG2a **(E)**, and IgG:IgM ratio **(F)**. The data for days 14 and 28 are presented as means with the standard error of the mean, and inserts show individual data for each mouse at day 7 with boxes showing the 25-th and 75-th percentile, the median, and whiskers between minimum and maximum points. Significance was calculated by unpaired Student 2-tailed *t*-test and the results are represented by stars (*p < 0.05, **p < 0.01, ***p < 0.001). Statistically significant fold-differences between the means in unadjuvanted and adjuvanted groups vaccinated by the same delivery route observed at day 28 are indicated on each panel. **(G)** Vaccine-specific IgG2:IgG1 ratio measured at day 7 post-vaccination. **(H)** HAI titers measured against A/California 07/09 H1N1 virus at day 28 post-vaccination. The titers below the detection level 10 were assigned a titer of 5 for calculations and converted to log_2_ for statistical analysis. Groups: gray—naïve (*n* = 5), green—vaccine only (*n* = 9), blue—vaccine + 5 μg Quil-A (*n* = 4), black—vaccine + 5 μg cGAMP (*n* = 4).

We initially explored the effects of cGAMP or Quil-A administered with 1 μg of purified hemagglutinin (HA) of A/California 07/09 (H1N1) virus as a vaccine to evaluate candidate adjuvants in aged mice. The unadjuvanted vaccine was not protective: only 22% of vaccinated animals survived the challenge. ID-delivered cGAMP was previously reported to induce a strong Th-1 response in adult mice via activation of the STING pathway (29, 30). However, in our experiments all aged mice immunized ID with the vaccine supplemented with 5 μg cGAMP succumbed to infection upon challenge (Figures 1A,B). Quil-A alone, in a 5 μg dose, increased survival from 22 to 75% (Figure 1A) with ~14% maximal weight loss (Figure 1B). Compared with the unadjuvanted vaccine, the Quil-A – supplemented formulation induced a significant 10–30-fold increase in vaccine–specific antibody levels, while cGAMP alone induced 3–4-fold increase in IgG1 and IgG/IgM by day 14 (Figures 1C–F). The use of Quil-A as adjuvant elicited an increase in the IgG2a level by seven fold detected as soon as day 7 of vaccination (Figure 1E), but the changes in the IgG2a/IgG1 ratios were not statistically significant between groups of vaccinated mice (Figure 1G), and the HAI titers remained mostly below the level of detection in all groups (Figure 1H). These data demonstrate that in aged mice, Quil-A alone is more effective than cGAMP alone at the concentrations tested, but neither adjuvant ensured complete protection against live virus challenge.

### Effect of Quil-A + cGAMP Combination in Aged Mice

**Figure 2:**
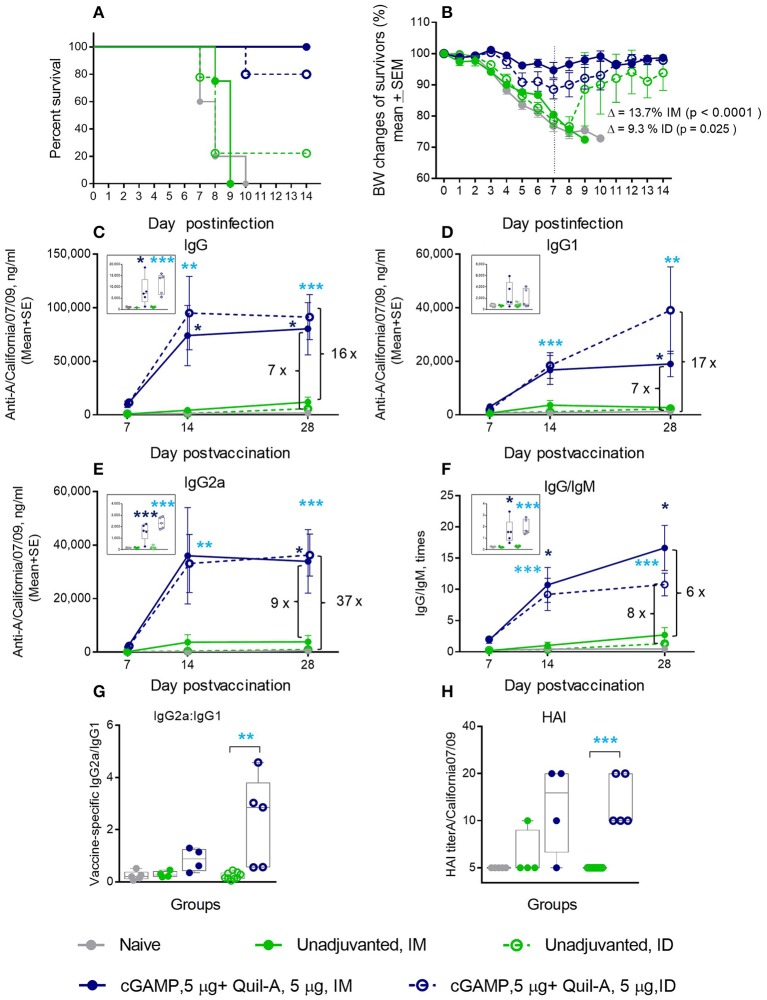
Synergetic effect of cGAMP and Quil-A adjuvants co-delivered with 1 μg of A/California 07/09 (H1N1) vaccine in aged mice. Challenge conditions and data panels correspond to those in Figure 1. **(A)** Survival and **(B)** weight chart of the surviving mice challenged with mouse-adapted influenza A/California 07/09 H1N1virus. Note significant differences in the average weight loss at day 7 postchallenge between unadjuvanted and adjuvanted groups delivered by the same route (ID or IM). **(C–F)**—time course of vaccine-specific antibody response plotted against day post-vaccination: IgG **(C)**, IgG1 **(D)**, IgG2a **(E)**, and IgG:IgM ratio **(F)**. Significance was calculated by Student 2-tailed *t*-test and the results are represented by stars (*p < 0.05, **p < 0.01, ***p < 0.001). **(G)** Vaccine-specific IgG2:IgG1 ratio measured at day 7 post-vaccination. **(H)** HAI titers measured against A/California 07/09 H1N1 virus at day 28 post-vaccination. Groups: gray—naïve (*n* = 5), green solid lines and filled circles—vaccine only IM (*n* = 4), green broken lines and empty circles—vaccine only ID (*n* = 9), blue solid lines and filled circles –vaccine adjuvanted with 5 μg cGAMP + 5 μg Quil-A IM (*n* = 4), blue broken lines and empty circles –vaccine adjuvanted with 5 μg cGAMP + 5 μg Quil-A, ID (*n* = 5).

We immunized aged mice with the same vaccine adjuvanted with a combination of 5 μg of each compound by ID or IM injections and observed that survival of the ID-immunized animals increased from 22 to 80%, with a 12% average weight loss after challenge. When this formulation was delivered IM, we observed a remarkable improvement in survival from zero to 100%, and the average maximal weight loss was as low as 5% in this group (Figures 2A,B). All isotypes of vaccine-induced antibodies increased to a greater extent than was observed with the individual adjuvants (compare panels C-E in Figures 1, 2). In particular, the levels of IgG2a isotype antibodies exhibited a 10–15-fold increase on day 7 post vaccination in the IM or ID groups, respectively, compared to the unadjuvanted vaccine delivered by the same route (insert on Figure 2E). The difference reached 93 fold in the ID group 1 week later. By day 28 the level of vaccine specific IgG2a rose slightly in the unadjuvanted groups, but it remained significantly higher in the adjuvanted groups (Figure 2E). A significant 10-fold increase in the vaccine-specific IgG2a/IgG1 ratio, indicative of a Th-1 shift in the immune response, was observed in the adjuvanted vs. non-adjuvanted ID group at day 7 of vaccination (*p* = 0.003, Student two-tailed *t*-test) and a ~3-fold increase (*p* = 0.051, Student two-tailed *t*-test) was detected between the corresponding IM groups (Figure 2G). Almost all aged mice in the Quil-A/cGAMP combination groups developed HAI titers of 10 or 20 by day 28 (Figure 2H). This substantial improvement in protection and functional antibody titers over non-adjuvanted vaccine exceeded the effects of the individual adjuvants, demonstrating a synergy between them.

### Comparison of Quil-A/cGAMP Combinations in Mature Adult vs. Aged Mice

**Figure 3:**
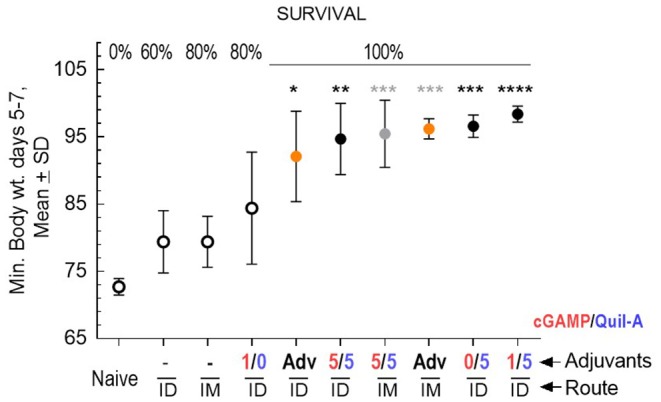
Protective efficacy of vaccination in adult mice, 5 per group except *n* = 9 in the naïve group, vaccinated with 1 μg of A/California 07/09 (H1N1) vaccine and challenged with 70 × LD_50_ of mouse-adapted A/California 07/09 H1N1virus. Average maximal weight loss relative to the pre-infection weight in each group between days 5–7 of challenge is plotted on the Y axis. Adjuvant composition and route of delivery for each group are indicated on the X axis; where a minus sign indicates unadjuvanted vaccine, AdV indicates AddaVax adjuvant, and numbers indicate the ratio of cGAMP (red) to Quil-A (blue), μg/μg. Empty circles represent groups with partial survival indicated by percentage on the top of the graph; filled circles represent groups with 100% survival. Yellow circles represent AddaVax—adjuvanted groups. Black and gray stars indicate level of statistical significance between unadjuvanted and adjuvanted ID and IM groups, respectively, as calculated by 1-way ANOVA with Tukey post-test (*p < 0.05, **p < 0.01, ***p < 0.001, ****p < 0.0001).

**Figure 4:**
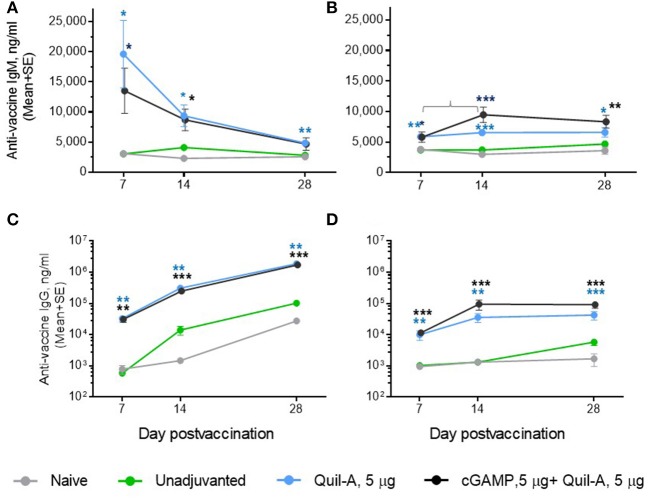
Comparison of the time course of antibody response between ID-vaccinated adult **(A,C)** and aged **(B,D)** mice. **(A,B)** vaccine-specific IgM; **(C,D)** vaccine-specific IgG. Note that in **(A,B)**, the Y scale is linear and in **(C,D)** it is logarithmic. Groups: gray—naïve (*n* = 5 in aged, 9 in adults); green—vaccine only (*n* = 9 in aged, 5 in adults); blue—vaccine + 5 μg Quil-A (*n* = 4 in aged, 5 in adults); black—vaccine + 5 μg Quil-A + 5 μg cGAMP (*n* = 5 in aged and in adults). The bracket in B denotes a 1.6—fold increase (*p* = 0.04) in vaccine-specific IgM level between days 7 and 14. Stars indicate significance levels of the differences between adjuvanted and non-adjuvanted group at the same time postvaccination calculated as described in Figure 1.

We challenged groups of ID or IM vaccinated mature adult mice with a 10-fold higher infectious dose compared to the aged animals, and ranked the groups by rate of survival and average weight loss (Figure 3). In spite of the high infectious dose, even those adult mice that received an unadjuvanted vaccine were partially protected, with 60 and 80% survival rates observed in the ID and IM groups, respectively, and all adjuvants in the doses tested except for 1 μg cGAMP completely prevented mortality. We did not observe differences in protection in the Quil-A/cGAMP combination group (5 μg each) delivered ID or IM (Figure 3). In the adult mice, the maximal geometric mean HAI titer 45.9 was detected in the 5 μg Quil-A group (Supplemental Figures S1A,B), while in the aged mice this was detected in the Quil-A/cGAMP combination groups using 5 μg of each (Figure 2H). Quil-A alone (5 μg) increased vaccine-specific antibody levels as effectively as in combination with 1–5 μg cGAMP (Supplemental Figure S2). A drop in the level of vaccine-specific IgM from day 7 to day 28 in mature adults (Figure 4A) was accompanied by a corresponding increase of vaccine-specific IgG (Figure 4C). The initial IgM response was 3–4 fold lower in the aged animals than in the adults (compare Figures 4A,B) and a 1.6-fold increase of vaccine-specific IgM in the Quil-A/cGAMP group was observed between days 7 and 14 (*p* = 0.04), but essentially remained at day 7 levels in the Quil-A group (Figure 4B). An increase in the level of vaccine-specific IgG was observed between days 7 and 14 in the aged animals (Figure 4D), but it was ~20 fold lower than observed in the adult mice by day 28 (Figure 4C). These data indicate that the adjuvant combination improved antibody class switching in the aged mice, but this process was significantly more efficient in the adult animals without use of an adjuvant.

### Mechanism of Potentiation of cGAMP Signaling by Quil-A

**Figure 5:**
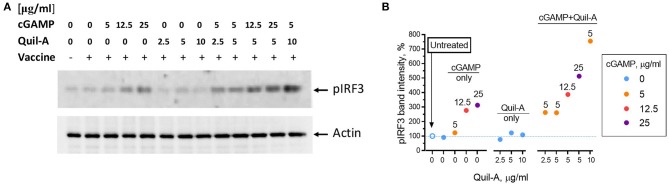
Phosphorylation of IRF3 in HeLa cells induced by Quil-A and cGAMP separately or in combination. **(A)** Representative western blot analysis of HeLa cell lysates prepared from cells treated for 1 h under conditions indicated for each lane. Concentration of vaccine was 5 μg/ml, concentrations of cGAMP and Quil-A varied from 0 to 25 μg/ml and from 0 to 10 μg/ml, respectively, as indicated for each condition. The original scans are presented in Supplemental Figure S6. **(B)** Intensity of the actin-normalized ~ 43 kDa pIRF3 band detected in the treated cell lysates relative to the non-treated cells (empty circle and dotted line correspond to first lane on the blot) is plotted against Quil-A concentration (X axis). Concentrations of cGAMP in μg/ml are color coded and shown over individual data points.

Binding of cGAMP to the STING adaptor protein triggers phosphorylation of the downstream factor IRF3 (36, 37). We compared the effect of each adjuvant alone or in combination on IRF3 phosphorylation in HeLa cells, which are known to respond to cGAMP (38). The cells were incubated with adjuvants for 1 hour, followed by assay of phosphorylated IRF3 levels in cell lysates by western blot (Figure 5A). Comparison of the intensities of the pIRF3 band normalized to actin showed that the addition of vaccine or Quil-A did not change pIRF3 levels, while cGAMP increased them up to 3-fold in a concentration-dependent manner (Figure 5B). A combination of Quil-A and cGAMP yielded the highest increase, about 8-fold, in pIRF3 levels as compared to untreated control. Notably, in the presence of 5 μg/ml cGAMP the increase in concentration of Quil-A from 5 to 10 μg/ml increased phosphorylation of IRF3 in HeLa cells six-fold (Figure 5B). Same experiments carried out in MEFs provided similar results and confirmed that phosphorylation of IRF3 was due to STING activation because it only occurred in STING+/+ but not in STING^−/−^ MEFs (Supplemental Figures S4, S5). These results support the conclusion that Quil-A enhances access of cGAMP to STING, and demonstrate that the combination of these compounds activates the IRF3 complex more effectively than cGAMP alone.

### Comparison With Current Approaches for Vaccination in Aged Humans

**Figure 6:**
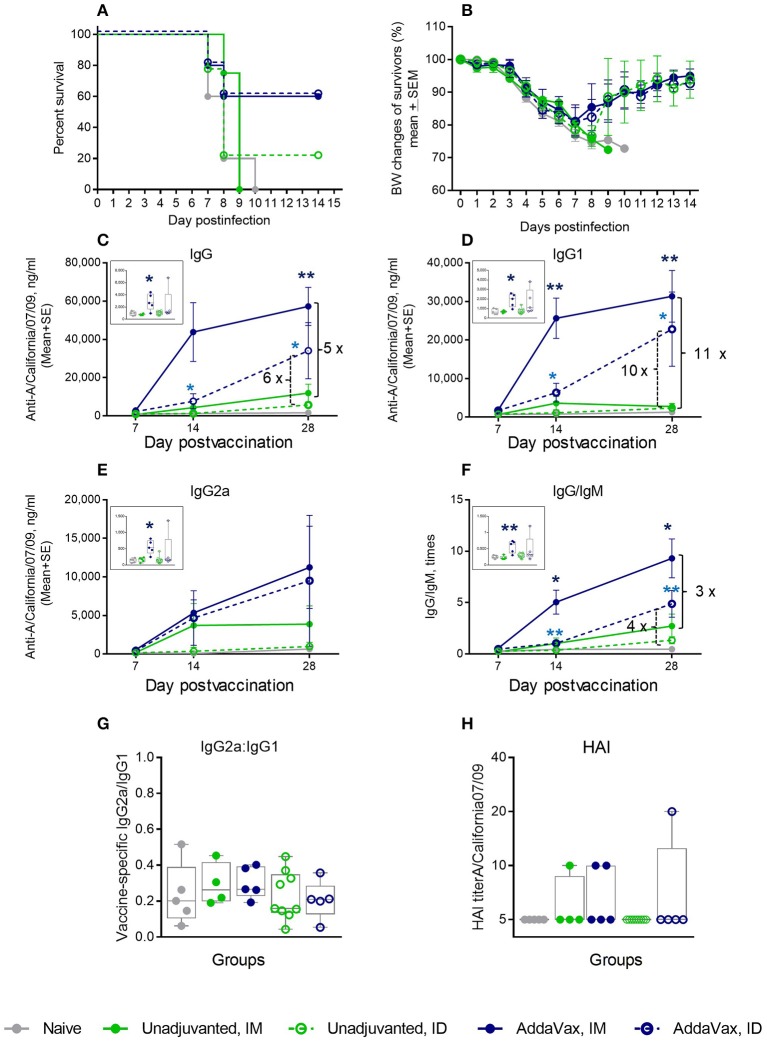
Effect of AddaVax adjuvant co-administered with 1 μg of A/California 07/09 (H1N1) vaccine in aged mice. Challenge conditions and data panels correspond to those in Figure 1. **(A)** Survival and **(B)** weight chart of the surviving mice challenged with mouse-adapted influenza A/California 07/09 H1N1virus; **(C–F)**—time course of vaccine-specific antibody response plotted against day post-vaccination: IgG **(C)**, IgG1 **(D)**, IgG2a **(E)**, and IgG:IgM ratio **(F)**. Significance was calculated by unpaired Student 2-tailed t-test and the results are represented by stars (*p < 0.05, **p < 0.01, ***p < 0.001). **(G)** Vaccine-specific IgG2:IgG1 ratio measured at day 7 post-vaccination. **(H)** HAI titers measured against A/California 07/09 H1N1 virus at day 28 post-vaccination. Groups: Gray—naïve (*n* = 5), green solid lines and filled circles—unadjuvanted vaccine IM (*n* = 4), green broken lines and empty circles–unadjuvanted vaccine ID (*n* = 9), blue solid lines and filled circles—vaccine + AddaVax IM (*n* = 5), blue broken lines and empty circles—vaccine + AddaVax ID (*n* = 5).

To determine whether a cGAMP/QuilA combination was more effective than the two currently used approaches for boosting the human immune response in aged patients, we carried out additional experiments. Aged mice were administered a single dose of 1 μg vaccine alone or in combination with a squalene-based adjuvant, AddaVax, which, according to the manufacturer instruction, is similar to the MF59 formulation used in humans. Addition of AddaVax increased survival after lethal challenge to 60% in both IM and ID groups (Figure 6A), but did not prevent high ~19% average weight loss at day 7 post challenge (Figure 6B). Consistent with previously reported data for a similar squalene-based adjuvant (39), the levels of vaccine-specific immunoglobulins were significantly elevated in the AddaVax groups as compared to the vaccine only groups (Figures 6C–E). The vaccine-specific IgG/IgM ratio in the AddaVax groups was also consistently higher than in non-adjuvanted groups (Figure 6F), indicating an increase in the efficiency of antibody class switch. We did not observe changes in the vaccine-specific IgG2a/IgG1 ratio (Figure 6G) that would indicate a change in the Th type of response. Although aged animals immunized IM with the adjuvanted formulation demonstrated slightly higher levels of vaccine-specific IgG and IgG1 and IgG/IgM ratio as compared to the ID-vaccinated mice, survival percentages were similar for both delivery routes. The aged mice developed very low HAI titers which were at or below the limit of detection in all groups in response to a single 1 μg vaccination dose (Figure 6H). In comparison, AddaVax effectively prevented mortality in vaccinated mature adult mice (4 month-old control, Supplemental Figure S3). Thus, although very effective in adults, AddaVax did not effectively prevent mortality or lessen morbidity in aged animals.

**Figure 7:**
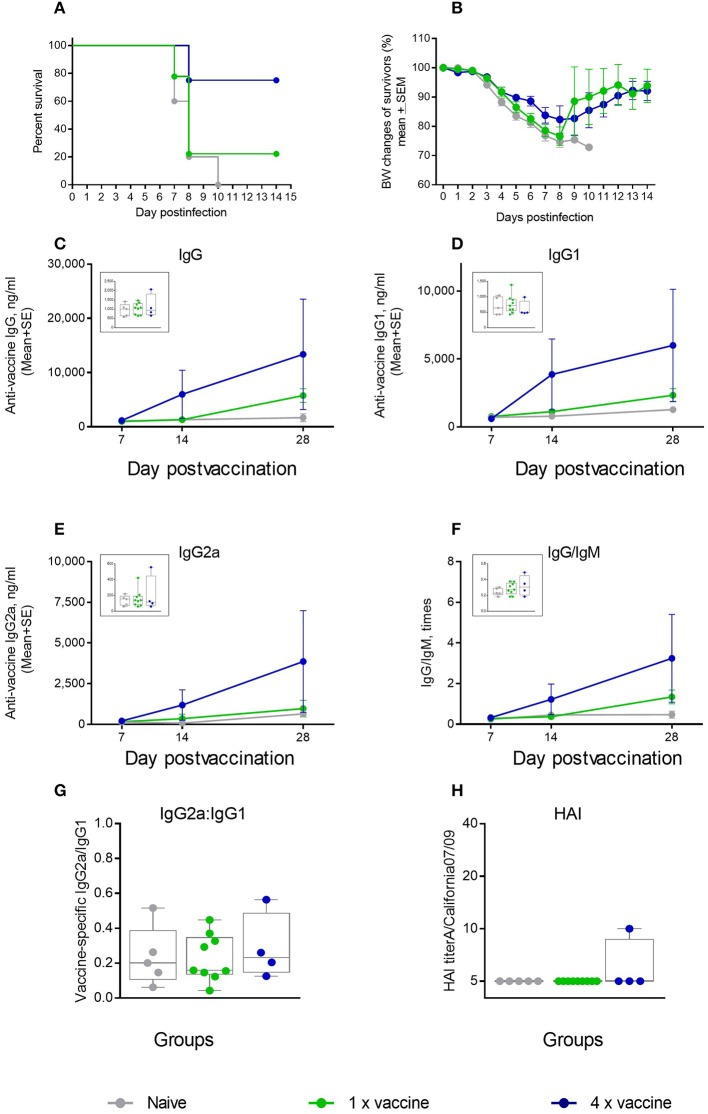
Effect of ID vaccination on protective immunity in aged mice using a 4-fold higher dose (4 μg) of vaccine antigen in comparison to the regular 1 μg dose. **(A)** Survival and **(B)** weight chart of the surviving mice challenged with the same virus; **(C–F)**—time course of vaccine-specific antibody response plotted against day of vaccination: IgG **(C)**, IgG1 **(D)**, IgG2a **(E)**, and IgG:IgM ratio **(F)**. The data for days 14 and 28 are presented as means with the standard error of mean, and inserts show individual data for each mouse at day 7 with the boxes showing the 25-th and 75-th percentile, the median, and whiskers between minimum, and maximum points. Statistically significant fold-differences between the means in unadjuvanted and adjuvanted groups vaccinated by the same delivery route observed for day 28 are indicated on each panel. **(G)** Vaccine-specific IgG2:IgG1 ratio at day 7 of vaccination. **(H)** HAI titers measured against A/California 07/09 H1N1 virus at day 28 of vaccination. Groups: gray—Naïve (*n* = 5), green—vaccine only (*n* = 9), blue −4 × vaccine (*n* = 4).

We further tested whether an ID vaccination with a 4-fold higher dose of an unadjuvanted antigen was protective in the aged mice. We observed that 75% of mice in the 4 μg dose group survived the challenge, compared to 22% survival in the 1 μg vaccine group (Figure 7A), but the maximal weight loss was as high as 16% (Figure 7B). No significant differences were observed in the levels of vaccine-specific IgG, IgG1, IgG2a, or in IgG/IgM and IgG2a/IgG1 ratios (Figures 7C–G) that would correlate with better survival in the 4 × antigen dose group, and HAI titers were mostly below the level of detection in both groups (Figure 7H). Thus, in aged mice, use of a 4-fold higher antigen dose yielded a comparable level of protection as that observed with the AddaVax adjuvant. In both cases, survival was improved, although not to 100%, but morbidity was not prevented as seen by significant weight loss observed in all groups after challenge. These data show that the current strategies used to vaccinate the aged population are also limited in their effectiveness in the aged mouse model. In particular, the improvement in protection and functional antibody titers over non-adjuvanted vaccine was reduced compared with the cGAMP/Quil-A combination, demonstrating the high potential of this adjuvant combination in overcoming the effects of immunosenescence. In this work we did not directly address T-cell responses. As T-cell immunity is important parameter of protection in the aged vaccines, future work is needed to define the effects of the novel adjuvant combination on this parameter.

## Discussion

The low protective efficacy of influenza vaccines in the aged population (40–43) is driving an intensive search for adjuvants that will elicit a more effective immune response (17, 44–46). Purified subunit vaccines such as used in this study are known for their good safety profile and low reactogenicity but also for relatively lower immunogenicity, especially in the aged, compared with whole inactivated virus. In the present study we evaluated a novel combination of a STING pathway agonist with a saponin adjuvant (Quil-A) in aged mice, a model that recapitulates responses to vaccination observed in aged humans. The criteria for protective immunity used in our study were survival and morbidity measured as weight loss after live virus challenge. The levels of the vaccine-specific immunoglobulins were also determined to estimate the differences in the magnitude of antibody response. The HAI titers, which are typically used as a correlate of protection (47), were almost undetectable in any of the aged animals, consistent with previously reported observations (17, 48), and thus not very useful.

We hypothesized that activation of the STING pathway, which is dysregulated during immunosenescence (49, 50), would enhance the innate immune response to vaccination due to a transient increase in the secretion of type I interferons. However, we did not observe an improvement in survival post challenge in aged mice immunized with the vaccine supplemented with free cGAMP (5 μg), although a modest increase in the vaccine-specific IgG/IgM ratio and IgG1 indicated that a low level of increase in antibody production did occur.

The cyclic dinucleotide cGAMP is a charged compound that does not freely penetrate the cell membrane, as is necessary for activation of the ER-resident STING. Thus, we further hypothesized that availability of cGAMP for the STING adaptor protein would be increased by the addition of a membrane-active compound to the vaccine formulation. We used Quil-A to permeabilize cell membrane and thus improve access of cGAMP to STING. Since in our work we used aged mice as a model for aged humans, we confirmed synergy between cGAMP and Quil-A in a human cell line to demonstrate the relevancy of our findings. In both human and mouse cells, the STING pathway was activated by a lower concentration of cGAMP in the presence of Quil-A than in its absence. Saponin was selected because in addition to enhancing membrane permeability for small molecules (51, 52) it is an effective adjuvant that stimulates production of proinflammatory cytokines and promotes cellular immune response to protein antigens. Separate studies with cGAMP (30) or Quil-A—containing adjuvants (53) were each reported to stimulate Th1 responses to influenza vaccines in adult mice. The rapid increase in the IgG2a antibody isotype in cGAMP/Quil-A adjuvanted groups that we observed was also consistent with a Th-1 bias of adjuvant action mode (54). We have found that in aged mice, cGAMP potentiated the protective immune response in the presence of Quil-A, while in adult mice Quil-A alone efficiently increased survival and HAI titers, and further addition of 1–5 μg cGAMP did not significantly increase the high protective efficacy of the A/California07/09 H1N1vaccine (55).

The protective immunity achieved in aged mice with the cGAMP/Quil-A adjuvant combination was demonstrated to be superior to that observed with a squalene-based adjuvant or high-dose antigen formulations, which are current treatment options used to better protect against influenza by vaccination of aged humans. Remarkably, the combination-adjuvanted vaccine provided 100% protection in the aged mice after a single vaccination. Our data also demonstrate that the cGAMP/Quil-A combination is effective as an adjuvant when co-delivered with the vaccine by either intradermal or systemic routes of administration, which is consistent with recently reported results for 3′3-cGAMP-loaded microparticles (56). Other adjuvant combinations and delivery vehicles containing STING agonists have been previously reported including use of aluminum hydroxide (57) and encapsulation in lipid nanoparticles (58) or microparticles (59, 60). Unlike these formulations, the cGAMP/Quil-A combination that we used is easily added to existing vaccines.

In conclusion, this study reports a novel combination adjuvant aimed at improving the protective response to current HA-based inactivated influenza vaccines, which is otherwise reduced as an effect of aging. Importantly, this combination adjuvant effectively increased the protective immunogenicity of a subunit vaccine in aged mice using a single immunization.

## Data Availability Statement

All datasets generated and analyzed for this study are included in article/Supplementary Material.

## Ethics Statement

The animal study was reviewed and approved by Institutional Animal Care and Use Committee at Emory University.

## Author Contributions

EV and RC designed the experiments, wrote, and edited manuscript. EV and DT performed experiments. EV processed data and prepared figures.

### Conflict of Interest

The authors declare that the research was conducted in the absence of any commercial or financial relationships that could be construed as a potential conflict of interest.
