# Supplementary material for: Combination of STING Pathway Agonist With Saponin Is an Effective Adjuvant in Immunosenescent Mice
Source: Front Immunol. 2019 Dec 23;10:3006. doi: 10.3389/fimmu.2019.03006 (PMC6935580; doi:10.3389/fimmu.2019.03006)
Supplement: Supplementary file 1 [file Data_Sheet_1.pdf]

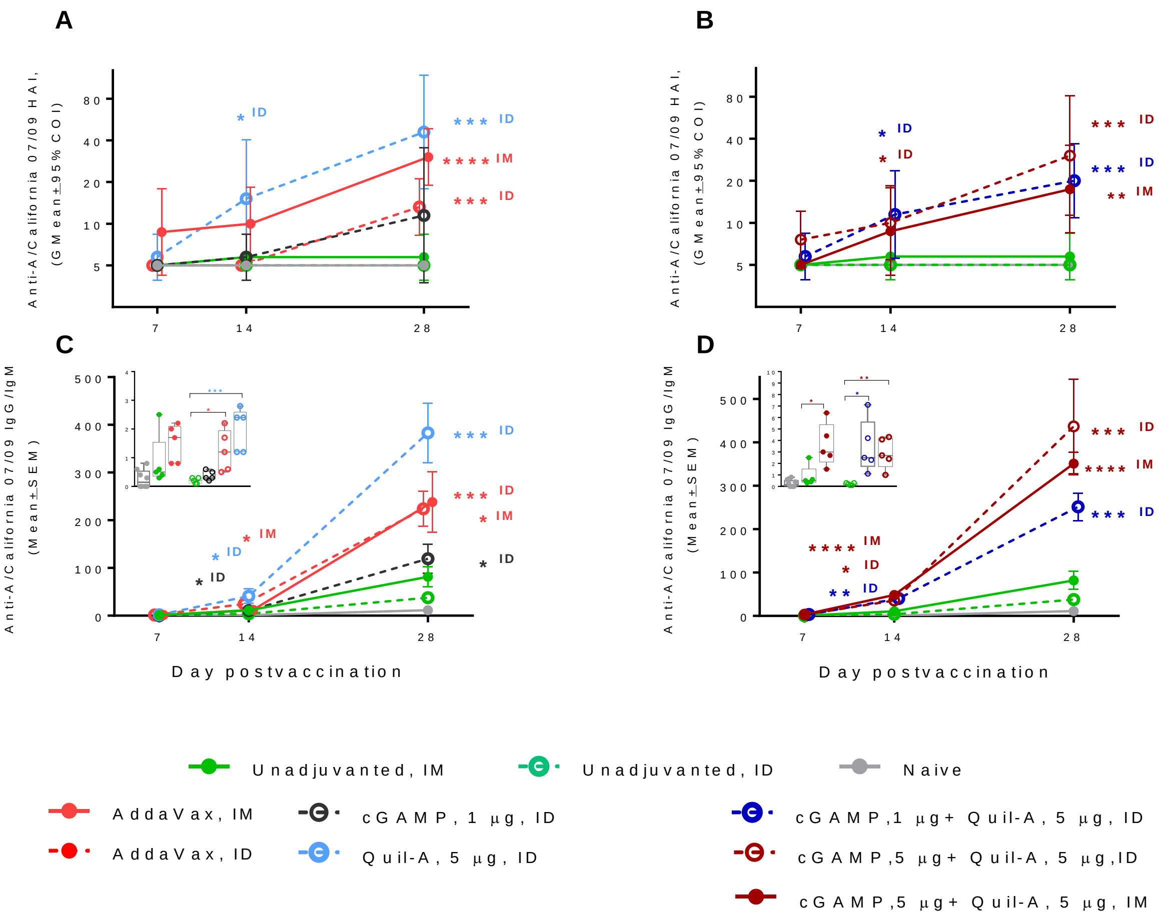

Supplemental Figure S1 Humoral response to the adjuvanted A/California 07/09 H1N1 vaccine in adult mice, n = 5 per group except n = 9 in Naïve group. Adjuvants were formulated with the vaccine either separately (A, C) or in combination (B, D). A, B – HAI titers. The titers below the detection level 10 were assigned a titer of 5 for calculations and converted to log2 for statistical analysis. C, D – ratio of vaccine-specific IgG/IgM. The solid lines and filled circles represent IM route of delivery, broken lines and empty circles – ID route of delivery. Groups: Green in all panels represent nonadjuvanted vaccine. On A and C: Black – cGAMP, 1 µg, Red – AddaVax, Light blue – Quil-A, 5 µg. On B and D: Dark blue - cGAMP, 1 µg + Quil-A, 5 µg; Red - cGAMP, 5 µg + Quil-A, 5 µg. The data for days 14 and 28 are presented as means with the standard error of the mean. The inserts in panels (C) and (D) show individual data for each mouse at day 7 with boxes showing the 25-th and 75-th percentile, the median, and whiskers between minimum and maximum points. The color-coded stars indicate the level of statistical significance (*p<0.05, **p<0.01, ***p<0.001, ****p<0.0001) between adjuvanted and nonadjuvanted IM and ID groups at the same time points calculated by Student 2-tailed t-test.

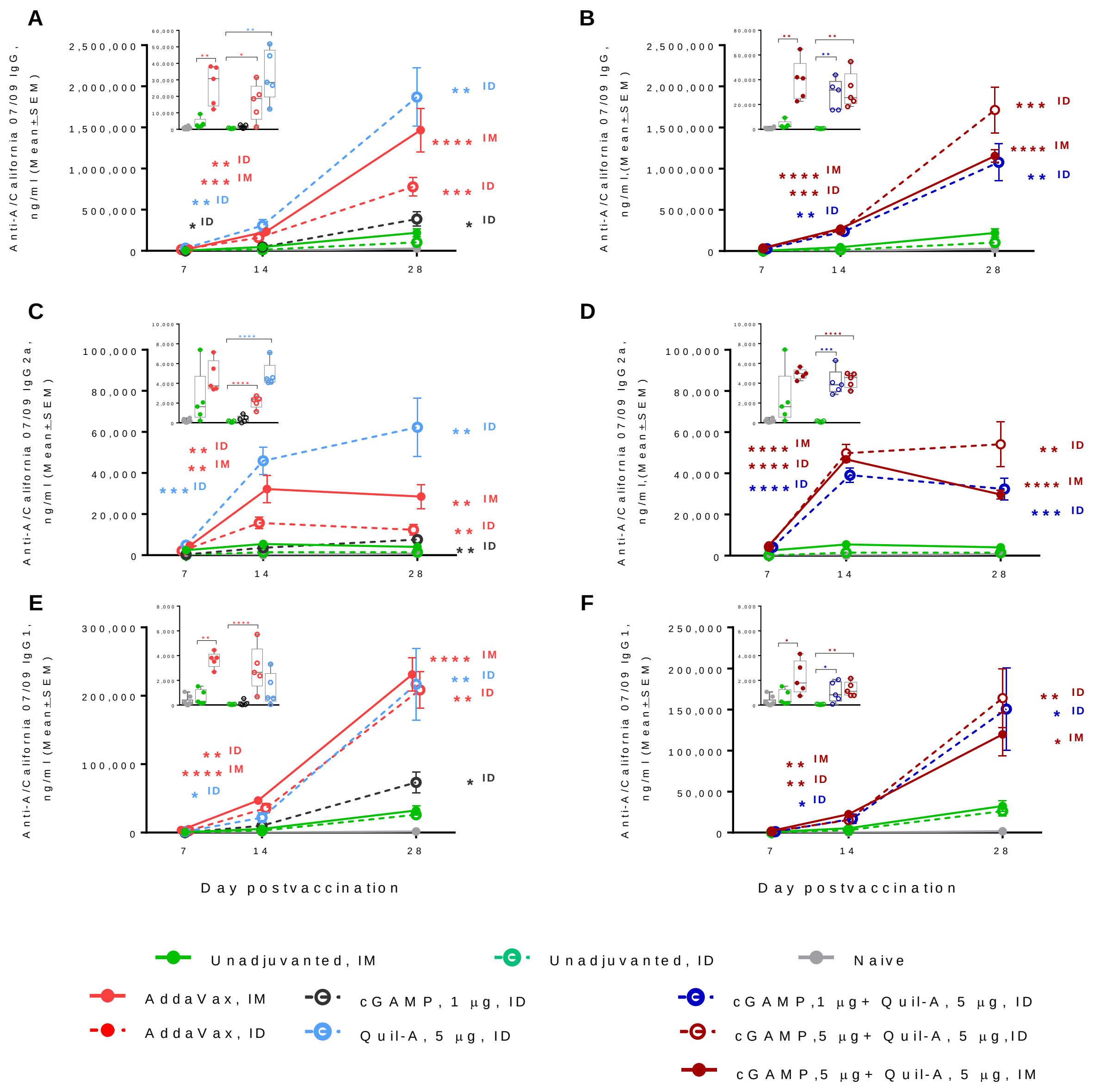

Supplemental Figure S2. Humoral response to the adjuvanted A/California 07/09 H1N1 vaccine in adult mice, n = 5 per group except n = 9 in Naïve group. Adjuvants were formulated with the vaccine either separately (A, C, E) or in combination (B, D, F). A, B – Vaccine-specific IgG; C, D – vaccine-specific IgG2a; E, F – Vaccine-specific IgG1. The solid lines and filled circles represent IM route of delivery, broken lines and empty circles – ID route of delivery. Groups: Green in all panels represent nonadjuvanted vaccine. On A, C, E: Black – cGAMP, 1 µg, Red – AddaVax, Light blue – Quil-A, 5 µg. On B, D, F – Dark blue - cGAMP, 1 µg + Quil-A, 5 µg; Red - cGAMP, 5 µg + Quil-A, 5 µg. The data for days 14 and 28 are presented as means with the standard error of the mean. The inserts in panels (C) and (D) show individual data for each mouse at day 7 with boxes showing the 25-th and 75-th percentile, the median, and whiskers between minimum and maximum points. The color-coded stars indicate the level of statistical significance (*p<0.05, **p<0.01, ***p<0.001, ****p<0.0001) between adjuvanted and nonadjuvanted IM and ID groups at the same time points postvaccination as calculated by Student 2-tailed t-test.

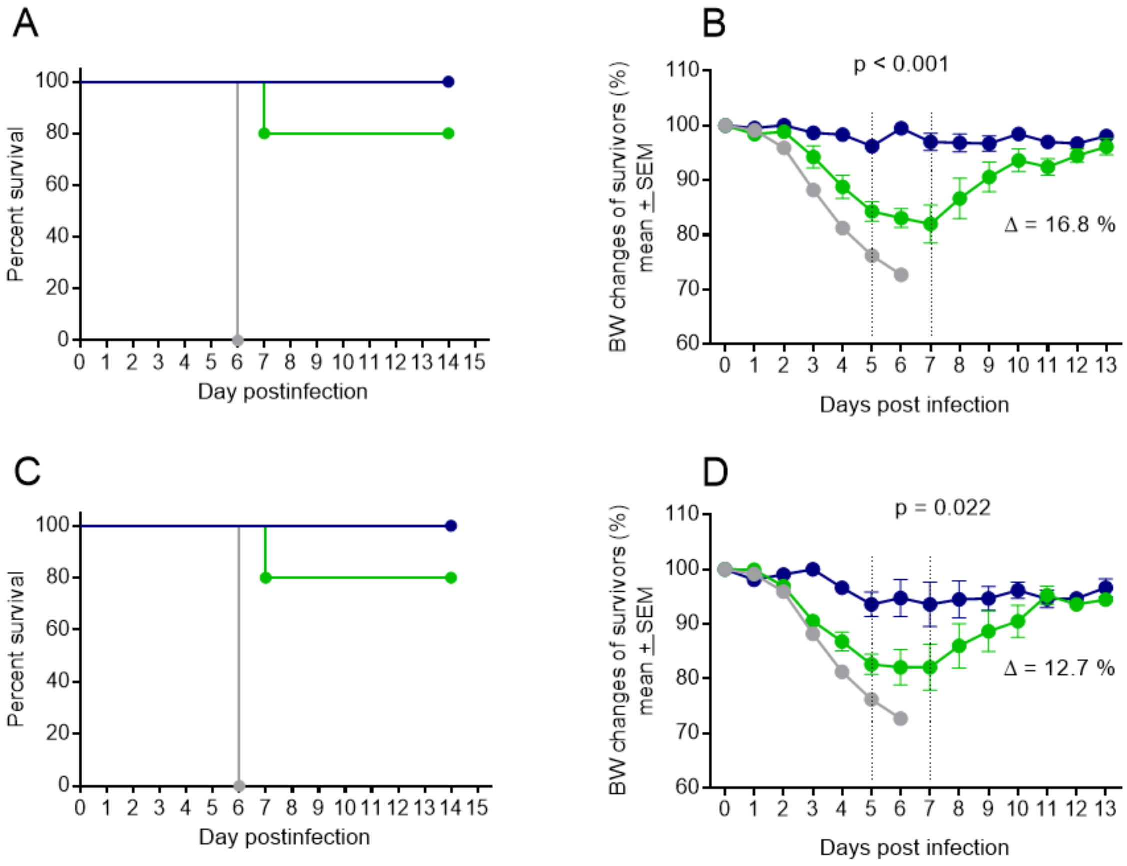

Supplemental Figure S3. Adjuvant effect of AddaVax adjuvant co-administered with H1N1 vaccine either by intramuscular injection (A, B) or ID (C, D) in adult mice, n = 5 mice in each group except n = 9 in Naïve group. A, C) Survival. B, D) weight loss of the surviving mice after challenge with 70 x LD_{50} of mouse-adapted A/California 07/09 H1N1 virus. P indicates significance level (Student 2-tailed t-test) of the difference in the mean maximal weight loss occurred between days 5 – 7 in adjuvanted (blue) and nonadjuvanted (green) groups.

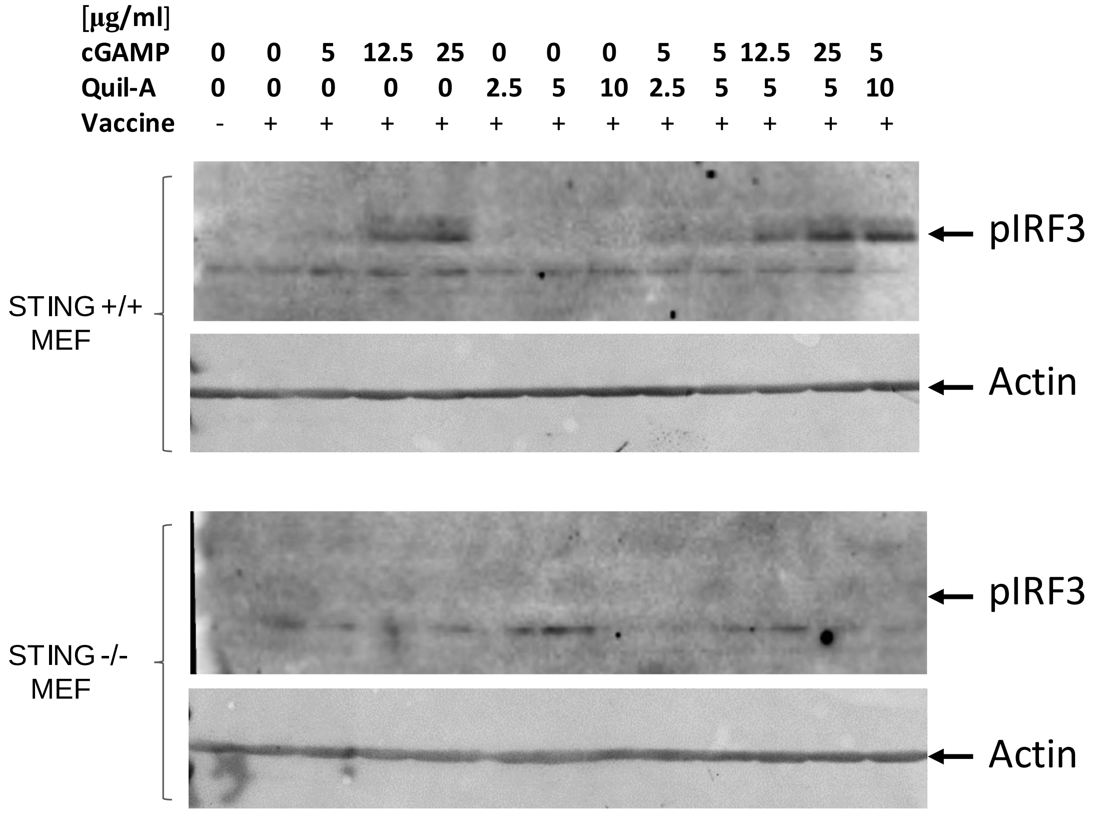

Supplemental Figure S4. Phosphorylation of IRF3 in mouse embryonic fibroblasts induced by Quil-A and cGAMP separately or in combination detected by western blot analysis. The wild type and STING-deficient MEFs were treated under conditions indicated for each lane for 1 hour and cell lysates were analyzed by western blot. Concentration of vaccine was 5 µg/ml, concentrations of cGAMP and Quil-A varied from 0 to 25 µg /ml and from 0 to 10 25 µg /ml, respectively, as indicated for each condition. The original scans are presented in supplemental figure S5.

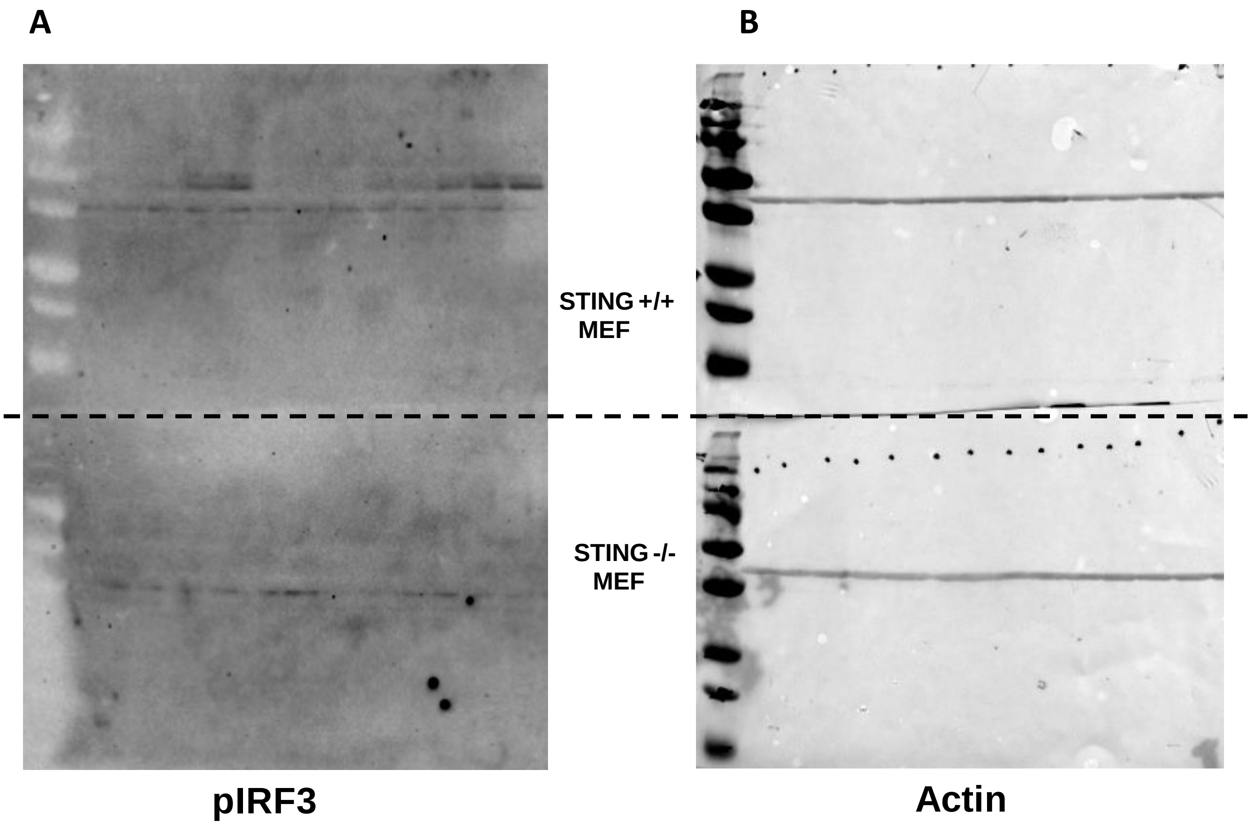

Supplemental Figure S5 Full scans of western blots presented in Figure S4. A) pIRF3 detection, B) Actin detected on the same membranes after striping.

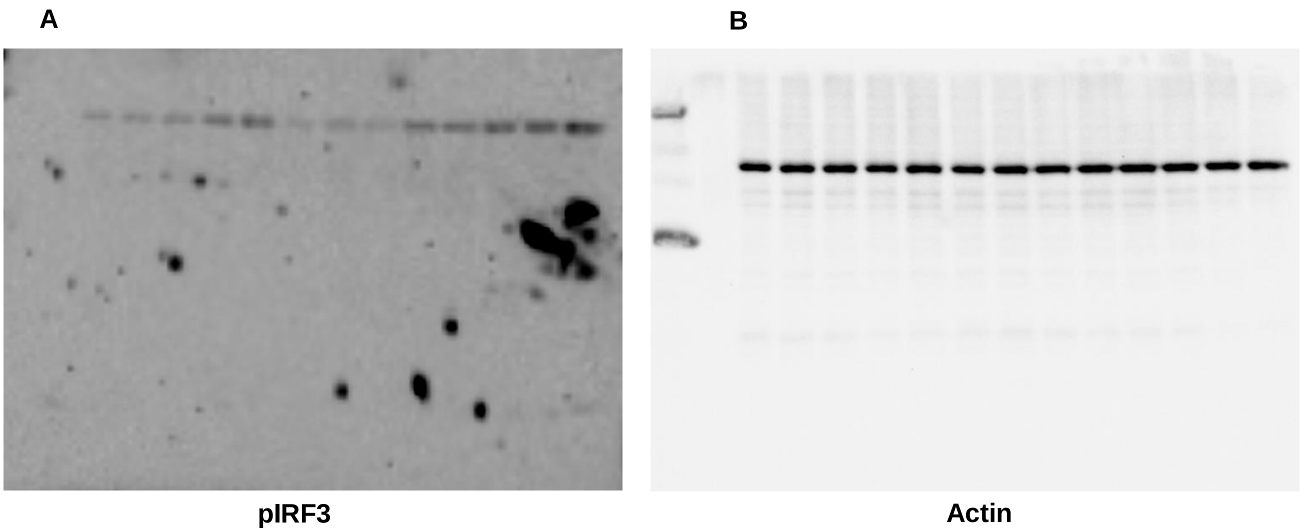

Supplemental Figure S6. Full scans of western blots presented in Figure 5. A) pIRF3 detection, B) Actin detected on the same membrane after striping.
